# Oral Treatment with the Vimentin-Targeting Compound ALD-R491 Mitigates Hyperinflammation, Multi-Organ Injury, and Mortality in CLP-Induced Septic Mice

**DOI:** 10.3390/life15101563

**Published:** 2025-10-06

**Authors:** Jianping Wu, Shuaishuai Wang, Kuai Yu, Zijing Xu, Xueting Wu, Deebie Symmes, Lian Mo, Chun Cheng, Ruihuan Chen, Junfeng Zhang

**Affiliations:** 1 Laboratory Animal Center, Nanjing University of Chinese Medicine, Nanjing 210023, China; wujianping@njucm.edu.cn; 2 School of Medicine, Nanjing University of Chinese Medicine, Nanjing 210023, China; 3 Aluda Pharmaceuticals, Inc., Union City, CA 94587, USA; 4 Luoda Biosciences, Inc., Chuzhou 239234, China

**Keywords:** sepsis, vimentin, host-directed therapy, CLP model, small molecule, in vivo efficacy

## Abstract

Sepsis is a life-threatening condition driven by a dysregulated host response to infection, with high mortality and few treatment options. Decades of failed drug development underscore the urgent need for therapies with novel mechanisms of action. Vimentin, an intermediate filament protein, acts as a network hub that senses and integrates cellular signals. Its involvement in key sepsis pathologies, including infection, hyperinflammation, immunosuppression, coagulopathy and metabolic dysregulation, positions it as a potential therapeutic target. This study evaluated the efficacy of ALD-R491, a novel small-molecule vimentin modulator, in a murine model of polymicrobial sepsis induced by cecal ligation and puncture (CLP). Mice received ALD-R491 prophylactically or therapeutically, alone or with ceftriaxone. The treatment significantly reduced serum levels of key biomarkers of sepsis, including C-reactive protein (CRP), lactate (Lac), tumor necrosis factor-alpha (TNF-α) and interleukin-6 (IL-6), and dose-dependently improved the survival of septic mice. Organ-specific analysis confirmed the effects of ALD-R491 in mitigating hyperinflammation and multi-organ injury. The treatment reduced pulmonary edema and inflammation; preserved liver tissue architecture and improved hepatic function with lowered alanine aminotransferase/aspartate aminotransferase (ALT/AST); decreased kidney tubular damage; and improved renal function with lowered creatinine/blood urea nitrogen (BUN). These preclinical findings indicate that the vimentin-targeting agent ALD-R491 represents a promising therapeutic candidate for sepsis and merits further clinical investigation.

## 1. Introduction

Sepsis is life-threatening organ dysfunction caused by a dysregulated host response to infection [1]. In the US, there are over 1.7 million sepsis cases in adults each year resulting in at least 350,000 deaths [2], with total estimated healthcare cost exceeding $62 billion annually [3], making it the most expensive hospitalization of any disease. Globally, there are approximately 48.9 million sepsis cases and 11 million deaths each year, with mortality rate over 20% [4]. Recognizing its severity, the World Health Organization (WHO) has designated sepsis as a top global health priority [5].

Despite decades of research, no drug targeting specific pathology of sepsis has demonstrated definitive efficacy in clinical trials [6]. In clinic, sepsis treatments remain largely supportive, including fluid resuscitation, vasopressors, and organ support, in addition to broad spectrum antibiotics. Although sepsis originates from a microbial infection, its high mortality and severe complications result from a dysregulated and unpredictable progression through hyperinflammation, immunosuppression, coagulopathy, and systemic metabolic collapse [7]. An effective treatment must therefore simultaneously target multiple key components of this multifaceted and often conflicting pathogenesis.

Vimentin is a highly conserved intermediate filament protein abundantly expressed in mesenchymal cells, such as fibroblast and immune cells. While non-essential under normal conditions [8], it plays a critical role in numerous pathological processes [9]. As a key regulator of infection and host response [10], vimentin serves as a central node for multiple cellular processes relevant to sepsis, including microbial infection and clearance [11,12,13], inflammation and immunosuppression [14,15,16], and coagulation and metabolism [15,17,18,19]. Vimentin-knockout mice exhibit reduced lung injury and improved survival following challenge with LPS, a microbial mediator of sepsis and systemic inflammation [14]. In sepsis patients, circulating vimentin levels are significantly elevated and correlate with hospital mortality [10,20], suggesting that vimentin is a promising therapeutic target for sepsis.

The novel small-molecule compound ALD-R491 was identified as a specific binder of vimentin through affinity-based proteomic profiling in live cells [21]. Binding of ALD-R491 alters key physical properties of vimentin intermediate filaments (VIFs), including flexibility, stability, and solubility [22,23]. Unlike conventional drug targets, vimentin has no intrinsic enzymatic activity. Its regulatory role as a coordinator or signal integrator [24,25] arises from the physical interactions of VIFs with a wide array of proteins that normally serve physiological functions but can become pathogenic in disease. These interactions underlie the diverse pathogenic roles linked to vimentin. By reducing VIF flexibility and presumably limiting VIF conformational diversity, ALD-R491 decreases the capacity of vimentin to engage with its binding partners [23]. Consistent with this mechanism, ALD-R491 has been shown to remodel the vimentin interactome [26], reduce the motility of cells and the release of intercellular communication mediator exosomes [27], and exert host-directed anti-bacterial, anti-viral, and anti-inflammatory effects [23,28], all of which are closely relevant to sepsis pathology.

This study evaluates ALD-R491’s therapeutic potential in sepsis using the cecal ligation and puncture (CLP) model, which closely mimics clinical polymicrobial sepsis [29]. We assess its impact on hyperinflammation, organ injury, disease severity, and survival, aiming to determine whether vimentin modulation offers a viable therapeutic approach for sepsis.

## 2. Materials and Methods

### 2.1. Compound and Purity Quotient

The vimentin-binding compound used in this study was ALD-R491, (E)-1-(4-fluorophenyl)-3-(4-(4-(morpholine-1-yl)-6-styryl-1,3,5-triazinyl-2-amino)phenyl) urea [27]. It has a molecular formula of C_28_H_26_FN_7_O_2_, a molecular weight of 511.55, and a purity of ≥98% (HPLC). The compound was synthesized at Bellen Chemistry Co., Ltd., Beijing, China, analyzed at Porton Pharma Solutions, Ltd., Chongqing, China, and provided by Luoda Biosciences, Inc., Chuzhou, China. ALD-R491 is a highly stable small-molecule compound with excellent physical and chemical characteristics. This compound remained stable under high temperature (60 °C), high humidity (92.5%RH), and accelerated conditions (40 °C/75%RH) in a stress testing. Its projected ambient shelf life exceeds 5 years. The compound was formulated for in vivo experiments with vehicle solution containing 10% citric acid (Solarbio, Beijing, China) and 2% SDS (Solarbio, Beijing, China).

### 2.2. Animals

SPF male C57BL/6J mice (18–20 g) aged 6–8 weeks were purchased from Shanghai SLAC Laboratory Animal Co., Ltd. (Shanghai, China) and housed in the Laboratory Animal Center of Nanjing University of Chinese Medicine. The animal facility was maintained at a temperature between 20 °C and 25 °C and a relative humidity between 40% and 70% on a regular light–dark cycle. The mice had free access to food and water. All experimental procedures were approved by the Animal Ethics Committee of Nanjing University of Chinese Medicine (No. 202306A021).

### 2.3. Drug Treatment and Groups

Prophylactic Protocol: Mice were divided into four groups for survival endpoints and four groups for inflammation and tissue injury analysis: sham surgery + vehicle, CLP + vehicle, CLP + ALD-R491 L (0.2 mg/kg), and CLP + ALD-R491 H (1 mg/kg). The selection of the ALD-R491 dose was based on the results of our previous research [23,28] and a pilot dose-finding experiment using CLP model. Treatments were administered 10 min before CLP surgery (day 0). In the survival study (Protocol 1), vehicle or ALD-R491 (at different doses) was given via oral gavage once daily for six consecutive days from day 1 to day 6. In the inflammation and tissue injury study (Protocol 3), treatments were administered 10 min before surgery and again 16 h post-surgery.

Therapeutic Protocol: Mice were divided into five groups for survival endpoints (Protocol 2): sham + vehicle, CLP + vehicle, CLP + ceftriaxone (50 mg/kg), CLP + ALD-R491 L (0.2 mg/kg) + ceftriaxone (50 mg/kg), and CLP + ALD-R491 H (1 mg/kg) + ceftriaxone (50 mg/kg). Treatments were given 2 h post-CLP surgery (day 0). Vehicle, ALD-R491, and ceftriaxone were then administered via oral gavage once daily from day 1 to day 6.

### 2.4. Cecal Ligation and Punction-Induced Mice Sepsis

Sepsis was induced using the standard cecal ligation and puncture (CLP) procedure. Briefly, mice were anesthetized via intraperitoneal injection of tribromoethanol (0.2 mL/10 g). A midline incision was made to expose the cecum, which was tightly ligated 1 cm from its distal end with a 3–0 silk suture and punctured once with a 21-gauge needle. A small amount of stool was gently extruded before repositioning the cecum into the abdominal cavity. The abdomen was closed with simple interrupted sutures, and the skin was sealed with tissue glue (3M Company, Maplewood, MN, USA).

For fluid resuscitation, 1 mL of saline was administered subcutaneously. Sham-operated mice underwent laparotomy without CLP. Survival analysis was conducted by monitoring mortality for seven consecutive days.

### 2.5. Disease Severity Evaluation

Disease severity in each mouse was assessed based on self-grooming, mobility, body position, and body weight loss [30]. Self-grooming: 0 = normal; 1 = dull/matte fur; 2 = ruffled/bristled fur. Mobility: 0 = normal (mobile without stimuli); 1 = reduced (less responsive to stimuli); 2 = immobile (unresponsive to stimuli). Body position: 0 = normal (fully extended); 1 = hunched posture; 2 = lying on the side. Body weight loss: 0 = minimal (<10%); 1 = moderate (10–14.9%); 2 = severe (15–19.9%). Weight loss in sham controls was adjusted to account for surgical effects.

A composite disease severity score was calculated by summing all category scores. Mice that died received the maximum score (8) from the day of death. Scoring classification: 0–2 = healthy; 3–5 = moderate disease; 6–8 = severe disease. Disease severity was scored by an assessor blinded to the treatment allocations.

### 2.6. Evaluation of Lung Edema

Lung edema was assessed using the wet-to-dry weight ratio. Tissue from the superior lobe of the right lung was dissected, blotted with filter paper, and weighed to obtain the wet weight. It was then dried at 65 °C for 48 h to determine the dry weight. The wet/dry weight ratio was calculated accordingly.

### 2.7. Systemic Inflammation Analysis

To measure serum inflammatory cytokine levels, mice were anesthetized with isoflurane, and blood samples were collected from the orbital venous plexus. The samples were centrifuged at 3500 rpm for 20 min at 4 °C, and the serum was stored at −80 °C. Tumor necrosis factor-alpha (TNF-α) (BioLegend, San Diego, CA, USA), interleukin-6 (IL-6) (BioLegend, USA), and C-reactive protein (CRP) were quantified using ELISA kits (RuixinBio, China).

### 2.8. Measurement of Common Biomarkers for Sepsis and Tissue Damage

Metabolic stress and systemic tissue damage markers lactate (Lac) and lactic dehydrogenase (LDH), liver injury markers Alanine Aminotransferase/Aspartate Aminotransferase (ALT/AST), and renal function markers creatinine (CRE) and blood urea nitrogen (BUN) were analyzed using commercially available kits (Jiancheng Biotech, Nanjing, China).

### 2.9. Histological Examination

Mice in the tissue injury study were euthanized 24 h post-surgery. Fresh lung, liver, and kidney tissue samples were collected, fixed in 4% paraformaldehyde for at least 24 h, dehydrated, embedded in paraffin, and sectioned (4–5 μm thick). The sections were stained with hematoxylin and eosin (HE) and scanned using a Pannoramic DESK scanner (P-MIDI, 3D HISTECH, Budapest, Hungary).

Acute Lung Injury (ALI) Scoring: ALI severity was assessed blindly based on four parameters: hemorrhage, alveolar hyperemia, neutrophil infiltration, and alveolar space size [31]. Each parameter was scored from 0 to 4: 0 = Minimal; 1 = Mild; 2 = Moderate; 3 = Severe; 4 = Maximal.

Liver Injury Scoring: Liver injury was evaluated using a 0–4 scale based on hepatocyte necrosis (focal or confluent), inflammatory cell infiltration, hepatic sinusoidal congestion, ballooning degeneration, and microvascular thrombosis: 0 = No detectable injury; 1 = Minimal injury (<10% of hepatocytes affected); 2 = Mild injury (10–30% of hepatocytes affected); 3 = Moderate injury (31–60% of hepatocytes affected); 4 = Severe injury (>60% of hepatocytes affected).

Kidney Injury Scoring: Renal injury was assessed based on the proportion of damaged renal tubules, including brush border loss, tubular dilation/flattening, degeneration, cast formation, and vacuolization: 0 = No damage; 1 = <25% of tubules affected; 2 = 26–50% affected; 3 = 51–75% affected; 4 = >76% affected.

For each sample, 10 high-power fields (×400) were analyzed and averaged. All histopathological scorings were performed by a pathologist blinded to the treatment allocations.

### 2.10. Real-Time PCR Gene Expression Quantification

RT-PCR was used to quantify mRNA expression of *Interleukin-1β (IL-1β)*, *IL-6*, and *TNF-α* in lung tissues. Total RNA was extracted using the RNAprep Pure Tissue Kit (DP431, TIANGEN, Beijing, China) and Trizol reagent (Invitrogen, Waltham, MA, USA). cDNA synthesis was performed using the HiScript II One Step RT-PCR Kit (P612-01, Vazyme, Nanjing, China), with β-actin as the internal reference.

Primer sequences (Pishgam Biotech, Tehran, Iran) are *IL-1β* Forward GAAATGCCACCTTTTGACAGTG, Reverse TGGATGCTCTCATCAGGACAG; *TNF-α* Forward AAGGCCGGGGTGTCCTGGAG, Reverse AGGCCAGGTGGGGACAGCTC; *IL-6* Forward CCACTTCACAAGTCGGAGGCTTA, Reverse AGTGCATCATCGTTGTTCATAC. mRNA levels were quantified using SYBR Green 5 PCR Master Mix (TOYOBO, Osaka, Japan). The PCR conditions were as follows: 95 °C for 30 s (initial denaturation), 95 °C for 5 s, 60 °C for 31 s (40 cycles).

All reactions were performed in triplicate, and relative mRNA expression was calculated using the 2^−ΔΔ^Ct method.

### 2.11. Statistical Analysis

Statistical analyses were conducted using GraphPad Prism 6.0. Data were expressed as mean ± SEM or Min to Max. Survival curves were generated using the Log-rank (Mantel-Cox) test. Group comparisons were performed using: One-way ANOVA (for comparisons among >2 groups); Two-way ANOVA (for multiparametric analysis), followed by Tukey’s multiple comparisons test. A *p*-value of *p* < 0.05 was considered statistically significant.

## 3. Results

### 3.1. ALD-R491 Significantly Reduced Disease Severity and Mortality in Mice with CLP-Induced Sepsis

**Figure 1:**
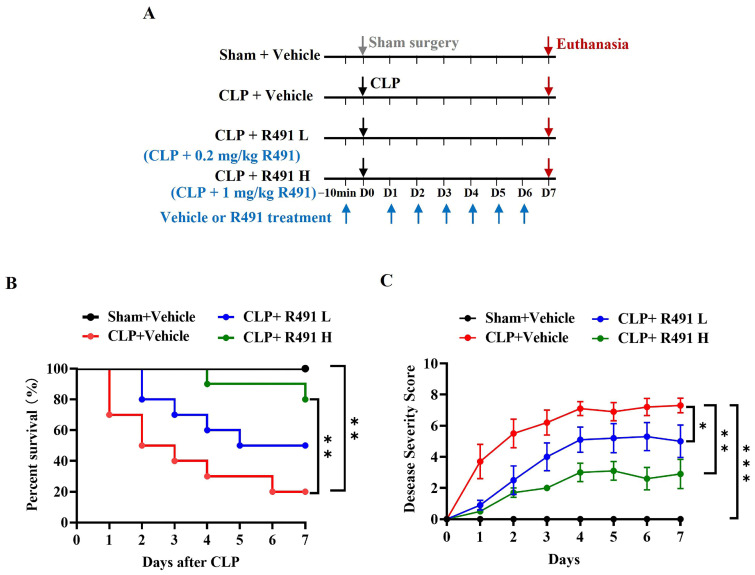
Effect of prophylactic administration of ALD-R491 on disease severity and mortality in septic mice. (**A**) The experimental scheme. C57BL/6J mice underwent either sham or CLP surgery, administrated by oral gavage with vehicle or ALD-R491 at low (L) or high (H) dose (0.2 mg/kg or 1 mg/kg) 10 min before the surgery on day 0 and once daily from day 1 to day 6, and terminated on day 7. The arrows indicate the dates of procedures performed (down arrow in gray: Sham surgery, down arrows in black: CLP surgery, down arrows in purple red: Euthanasia, up arrows in blue: Vehicle or R491 treatment). (**B**) The survival rate of mice within 7 days was calculated (*n* = 10 per group), ** *p* < 0.01 by Log-rank test. (**C**) Disease severity was scored each day for 7 days. Data are presented as the means ± SEM (the initial animal number in each group was 10 but decreased over the course of this study as death occurred), * *p* < 0.05, ** *p* < 0.01 and *** *p* < 0.001, two-way ANOVA.

To assess the therapeutic potential of the small-molecule vimentin modulator ALD-R491 in sepsis, we employed a cecal ligation and puncture (CLP)-induced sepsis model in mice, using mortality rate as the primary endpoint and disease severity as the secondary endpoint. Our previous study demonstrated that ALD-R491 reaches peak systemic exposure within 2 h [23]. Given that systemic proinflammatory cytokines peak around 6 h post-CLP, mice were treated 10 min before surgery to ensure drug action before the onset of a full-blown cytokine storm (Protocol-1, Figure 1A).

All mice in the sham group (surgery without CLP) survived (100%) throughout this study, exhibiting only mild, transient symptoms post-surgery. In contrast, half of the vehicle-treated CLP mice died within the first two days, with survival dropping to 20% by Day 7 (Figure 1B), accompanied by high disease severity scores (Figure 1C).

ALD-R491 treatment significantly delayed mortality, increasing 7-day survival rates to 50% at 0.2 mg/kg and 80% at 1 mg/kg (Figure 1B). Disease severity scores were also significantly lower in ALD-R491-treated mice at both doses compared to vehicle controls (Figure 1C). The dose-dependent improvement in survival and disease symptoms underscores ALD-R491’s therapeutic potential for sepsis treatment.

### 3.2. ALD-R491 Combined with Ceftriaxone Significantly Reduced Disease Severity and Mortality in Mice with CLP-Induced Sepsis

**Figure 2:**
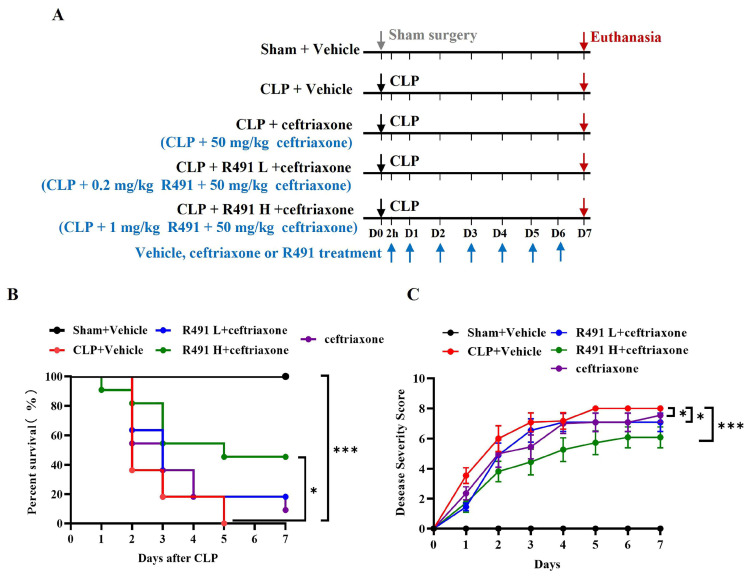
Effect of therapeutic administration of ALD-R491 on disease severity and mortality in septic mice. (**A**) The experimental scheme. C57BL/6J mice underwent either sham or CLP surgery, administrated by ALD-R491 (0.2 mg/kg or 1 mg/kg), ceftriaxone (50 mg/kg) or vehicle, 2 h after the surgery on day 0 and once daily from day 1 to day 6, and terminated on day 7. The arrows indicate the dates of procedures performed (down arrow in gray: Sham surgery, down arrows in black: CLP surgery, down arrows in purple red: Euthanasia, up arrows in blue: Vehicle or R491 treatment). (**B**) The survival rate of mice within 7 days was calculated (*n* = 10 per group), * *p* < 0.05 and *** *p* < 0.001 by Log-rank test. (**C**) Disease severity was scored each day for 7 days. Data are presented as the means ± SEM (the initial animal number in each group was 10 but decreased over the course of this study as death occurred), * *p* < 0.05, and *** *p* < 0.001, two-way ANOVA.

Ceftriaxone is a commonly used antibiotic for human sepsis; however, its administration alone often fails to achieve desired efficacy. To mimic clinical practice and further evaluate the therapeutic potential of ALD-R491, we administered it in combination with ceftriaxone. To allow this protocol to be closer to a therapeutic demonstration, the treatments started two hours after the CLP procedure (Protocol-2, Figure 2A). In this study, none of the vehicle-treated mice survived beyond five days post-CLP. In contrast, survival rates at Day 5 were 20% with ceftriaxone alone, and 20% and 50% when combined with 0.2 mg/kg and 1 mg/kg ALD-R491, respectively. By Day 7, survival rates were 10% (ceftriaxone alone), 20% (ceftriaxone + 0.2 mg/kg ALD-R491), and 50% (ceftriaxone + 1 mg/kg ALD-R491) (Figure 2B). Additionally, disease scores were lower in drug-treated groups compared to vehicle controls (Figure 2C). These findings indicate that while ceftriaxone alone provides minimal survival benefit in CLP-induced sepsis, its combination with ALD-R491 significantly improves survival.

### 3.3. ALD-R491 Significantly Reduced Systemic Inflammation and Cellular Injury in Septic Mice

**Figure 3:**
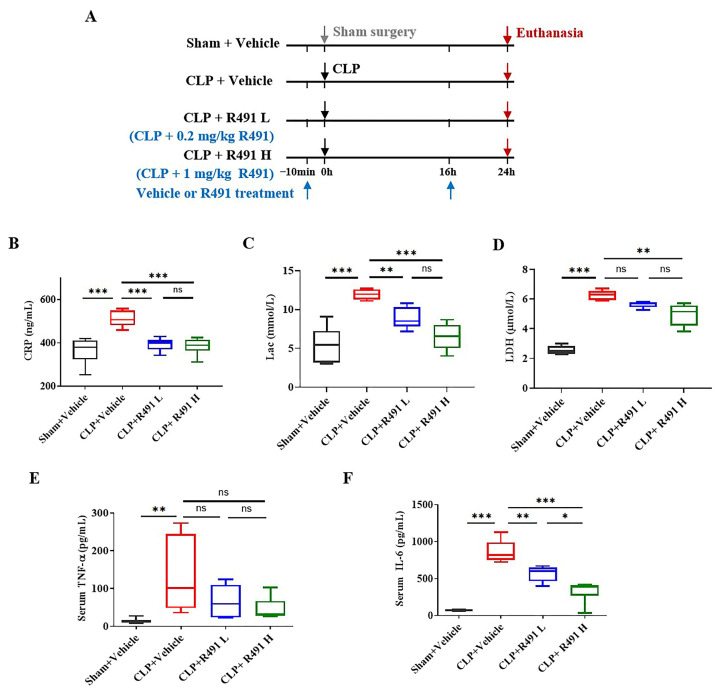
Effect of ALD-R491 on systemic inflammation in septic mice. (**A**) The experimental scheme. C57BL/6J mice underwent either sham or CLP surgery. At 10 min before surgery and 16 h after the surgery, the mice were treated with vehicle or R491 (0.2 mg/kg or 1 mg/kg) by oral gavage, and the samples were taken at 24 h post-surgery. The arrows indicate the dates of procedures performed (down arrow in gray: Sham surgery, down arrows in black: CLP surgery, down arrows in purple red: Euthanasia, up arrows in blue: Vehicle or R491 treatment). (**B**–**F**) Concentrations of CRP (**B**), Lac (**C**), LDH (**D**) TNF-α (**E**), and IL-6 (**F**), in serum at 24 h after CLP procedure. Data are presented as Min to Max (*n* = 6 per group), ns (no significance), * *p* < 0.05, ** *p* < 0.01 and *** *p* < 0.001, one-way ANOVA.

With the confirmed efficacy of ALD-R491 in reducing symptoms and mortality of mice with CLP, we ran a separate in vivo study designed to understand the effects of the compound on hyperinflammation, especially on biomarkers and cytokines that are predominant in this model as well as in human sepsis. The mice were given treatment with vehicle or ALD-R491 at different doses at 10 min before and 16 h after the CLP (Protocol-3, Figure 3A). At 24 h post-surgery, the systemic levels of key biomarkers in sepsis significantly increased (Figure 3B–D), including C-reactive protein (CRP), an acute phase protein responding to infection and inflammation; lactate (Lac), an indicator of tissue hypoxia and metabolic stress; and lactate dehydrogenase (LDH), a maker of cellular injury, systemic inflammation and multiorgan dysfunction. The key proinflammatory cytokines in sepsis, IL-6 and TNF-α, were both significantly higher in the blood of mice with CLP than in mice with sham operation (Figure 3E,F). All these biomarkers were significantly reduced in mice treated with ALD-R491 at both low (0.2 mg/kg) and high (1 mg/kg) doses.

The serum CRP, Lac and TNF-α in the CLP mice treated with ALD-R491 at 1 mg/kg decreased significantly to a level that were comparable to their levels in the sham vehicle control mice (Figure 3B,C,E). LDH levels dose-dependently decreased in response to ALD-R491 treatment (Figure 3D). IL-6 was the most elevated cytokine (as high as 1000 times) in the serum of mice with CLP. ALD-R491 dose-dependently reduced the systemic levels of IL-6. The results indicate that CLP induced a sepsis in mice and ALD-R491 treatment significantly reduced systemic inflammation and cellular injury in septic mice.

### 3.4. ALD-R491 Significantly Alleviated Acute Lung Injury and Inflammation

**Figure 4:**
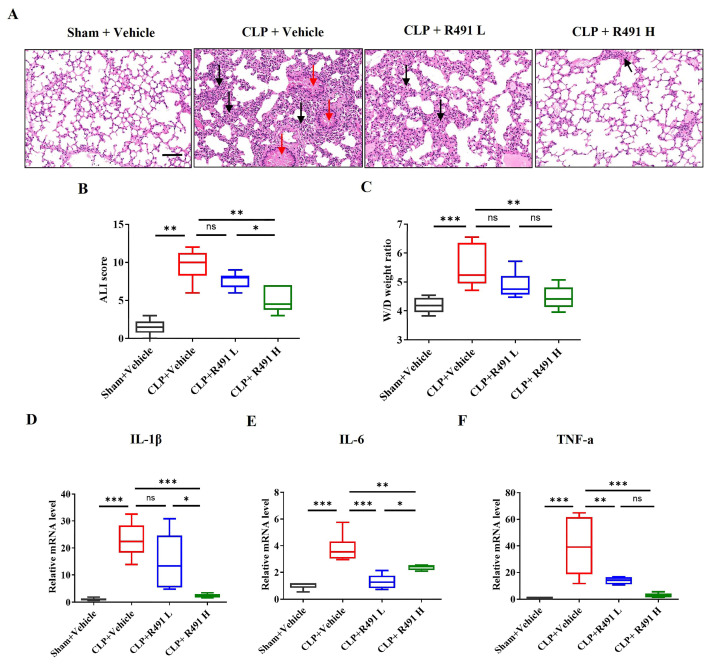
Effect of ALD-R491 on the histology and inflammation in lungs of septic mice. (**A**) Representative images of Sham mice and CLP mice treated with vehicle or ALD-R491 (1 mg/kg or 0.2 mg/kg). In CLP + Vehicle group, severe neutrophil infiltrations (black arrow) and congestion/edema (red arrow) were observed in the lung tissue. The CLP + ALD-R491 L group showed some alleviation of these conditions, while the CLP + ALD-R491 H group demonstrated significant mitigation of inflammatory injury. Scale bars: 100 μm. (**B**) Slides of H&E-stained lung tissues were scored semi-quantitatively according to the bleeding, alveolar hyperemia, neutrophil infiltration, and size of alveolar spaces to evaluate the lung histopathological damage. (**C**) Pulmonary edema was evaluated by the ratio of lung tissue wet-to-dry weight. (**D**–**F**) The levels of *IL-1β* (**D**), *IL-6* (**E**) and *TNF-α* (**F**) mRNAs in the lung tissues were quantified by RT-qPCR. Data are presented as Min to Max (*n* = 6–8 per group),ns (no significance), * *p* < 0.05, ** *p* < 0.01 and *** *p* < 0.001, one-way ANOVA.

The lung tissue from the Sham control group shows normal pulmonary architecture. The alveolar walls (septa) are thin, airspaces are clear and open, and there are minimal inflammatory cells within the interstitium. In contrast, the lung tissue from the CLP control group demonstrates significant features of acute lung injury (ALI). The normal lung structure is disrupted. The septa are markedly thickened due to edema (fluid accumulation) and infiltration by inflammatory cells. There is a substantial increase in inflammatory cells (likely neutrophils and macrophages) within the alveolar walls and potentially spilling into the airspaces (Figure 4A). Quantitative analysis shows a significantly higher ALI score in the CLP control group compared to the Sham control group (Figure 4B). The thickening of the septa encroaches upon and reduces the area of clear airspace. The potential presence of proteinaceous material in airspaces indicate edema, which is quantitatively supported by the increased Wet/Dry weight ratio (Figure 4C).

Compared to the CLP control group, the ALD-R491 low dose group appears less severe lung injury, although some signs of inflammation and structural changes are still present. The ALD-R491 high dose group shows a markedly improved lung tissue structure. The signs of inflammation, alveolar wall thickening, and overall tissue disruption are substantially reduced, suggesting a significant protective effect of the high dose of ALD-R491 against CLP-induced lung injury (Figure 4A). Quantitative analysis further demonstrates that the treatment with ALD-R491 significantly reduces CLP-induced acute lung injury scores and the lung wet-to-dry weight ratios in a dose-dependent manner, with the high dose offering greater protection than the low dose (Figure 4B,C).

The gene expression levels of key pro-inflammatory cytokines *IL-1β*, *IL-6* and *TNF-α* consistently demonstrate that CLP induces a significant inflammatory response in the lungs (Figure 4D–F). Treatment with ALD-R491 significantly attenuates this inflammatory response by dose-dependently reducing the expression of these cytokines. At 1 mg/kg, ALD-R491 reduced *IL-1β* and *TNF-α* expression in the lung tissue of CLP mice to the levels indistinguishable to those of sham vehicle controls (Figure 4D–F).

These results indicate that ALD-R491 treatment significantly reduces the acute lung injury and inflammation in the lung.

### 3.5. ALD-R491 Significantly Alleviated Acute Liver Injury and Inflammation

Effects of ALD-R491 on sepsis-associated hepatic injury were evaluated both histopathologically and biochemically.

**Figure 5:**
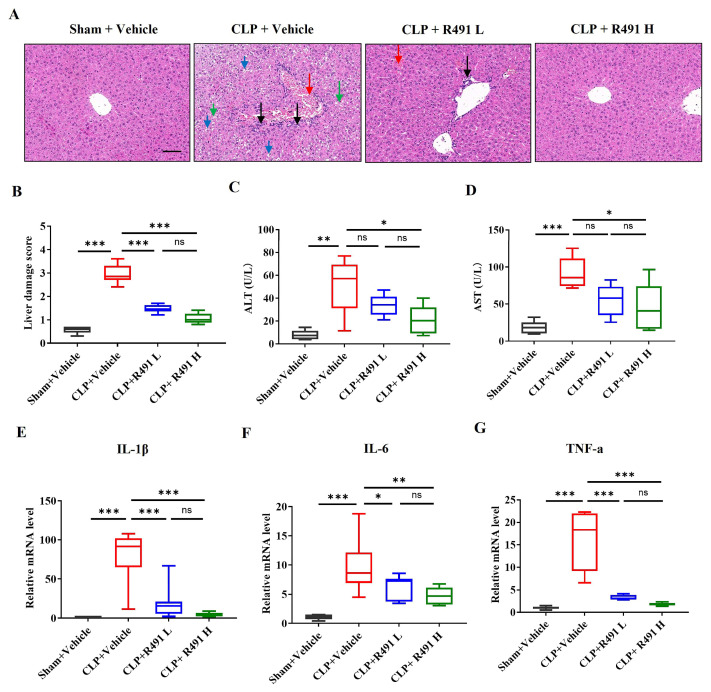
Effect of ALD-R491 on the histology and inflammation in liver of septic mice. (**A**) Representative images of Sham mice and CLP mice treated with vehicle or ALD-R491 (1 mg/kg or 0.2 mg/kg). The CLP control group exhibited vascular dilation and congestion (red arrow), hepatocyte injury (green arrow), cellular swelling and vacuolization (blue arrow), and an increased number of inflammatory cells (black arrow). The CLP + ALD-R491 L group showed some alleviation of liver tissue injury, while the CLP + ALD-R491 H group demonstrated significant mitigation of the damage. Scale bars: 100 μm. (**B**) Liver injury was evaluated based on hepatocyte necrosis (focal or confluent), inflammatory cell infiltration, hepatic sinusoidal congestion, ballooning degeneration, and microvascular thrombosis. (**C**,**D**) The levels of ALT and AST in the liver tissues were quantified by specific commercially available kits. (**E**–**G**) The levels of *IL-1β* (**E**), *IL-6* (**F**) and *TNF-α* (**G**) mRNAs in the liver tissues were quantified by RT-qPCR. Data are presented as Min to Max (*n* = 6–8 per group), ns (no significance), * *p* < 0.05, ** *p* < 0.01 and *** *p* < 0.001, one-way ANOVA.

The liver tissue from the Sham control group shows normal hepatic architecture. Hepatocytes are arranged in organized cords, sinusoids are clear, and there is minimal evidence of inflammation or vascular congestion (Figure 5A). In contrast, the liver tissue from the CLP control group displays significant pathological changes indicative of acute liver injury secondary to sepsis, including vascular congestion and erythrocyte stasis where sinusoids and larger vessels appear dilated and engorged with red blood cells, hepatocyte injury where the cells are swollen and vacuolized, the cord structures are disorganized, and inflammation where an increased number of inflammatory cells are visible within the sinusoids and potentially around portal tracts (Figure 5A). Quantitative assessment shows a significantly higher liver damage score in the CLP control group compared to the Sham control group (Figure 5B), indicating this CLP study presented a context of severe liver damage.

ALD-R491 treatment shows a clear dose-dependent improvement, which includes reduced vascular congestion and erythrocyte stasis, preserved liver architecture and hepatocyte organization, and decreased vascular congestion and immune cell infiltration (Figure 5A,B).

Consistent with the histological findings, ALD-R491 at both low and high doses significantly reduced CLP-induced elevations in serum levels of ALT and AST, established biomarkers of hepatic injury and metabolic stress (Figure 5C,D).

Intrahepatic cytokine analysis revealed significant CLP-induced upregulation of *IL-1β*, *IL-6*, and *TNF-α* gene expression relative to sham controls (Figure 5E–G). ALD-R491 treatment dose-dependently suppressed these pro-inflammatory cytokines, with the 1 mg/kg dose reducing levels near baseline.

### 3.6. ALD-R491 Significantly Alleviates Acute Kidney Injury and Restores Renal Function

**Figure 6:**
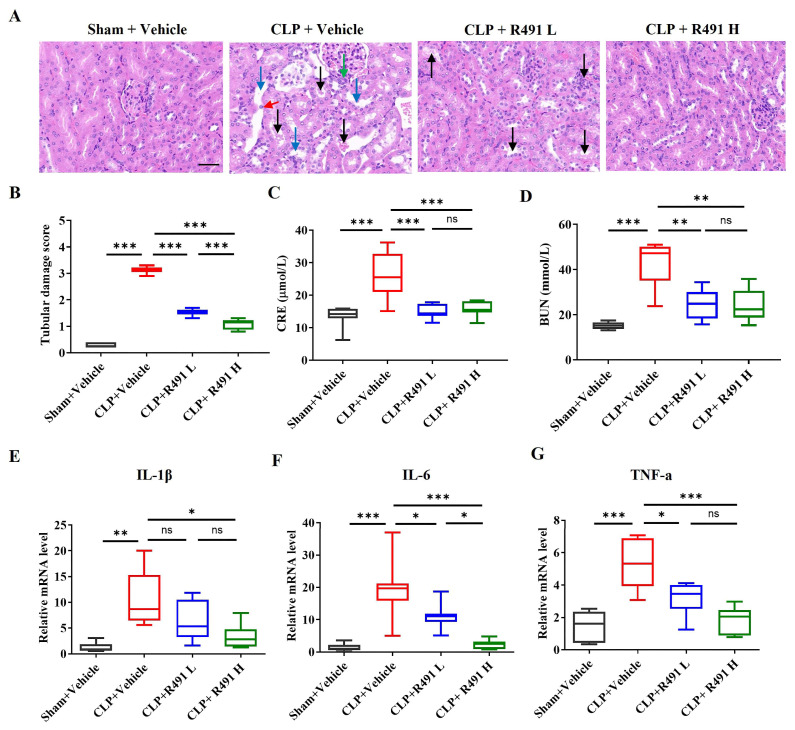
Effect of ALD-R491 on the injury in kidney of septic mice. (**A**) The collected kidneys were stained with hematoxylin and eosin (H&E). The CLP + Vehicle group exhibited changes such as renal tubular dilation (blue arrow), loss of brush border, casts (black arrow), cellular detachment (red arrow), and increased inflammatory cells (green arrow). The CLP + ALD-R491 L group showed some alleviation of renal tissue injury, while the CLP + ALD-R491 H group demonstrated significant mitigation of the damage. Scale bars: 50 μm. (**B**) Quantitative evaluation of morphological tubular damage. (**C**,**D**) The levels of CRE and BUN in the kidney tissues were quantified by specific commercially available kits. (**E**–**G**) The levels of *IL-1β* (**D**), *IL-6* (**E**) and *TNF-α* (**F**) mRNAs in the kidney tissues were quantified by RT-qPCR. Data are presented as Min to Max (*n* = 6–8 per group), ns (no significance), * *p* < 0.05, ** *p* < 0.01 and *** *p* < 0.001, one-way ANOVA.

The kidney tissue from the Sham control group displays relatively normal renal architecture. The glomeruli appear intact, and the tubules generally show well-defined lumens lined by healthy-looking cuboidal epithelial cells. The interstitial space between tubules is minimal, with few inflammatory cells (Figure 6A). In contrast, the kidney tissue from the CLP control group exhibits marked signs of acute kidney injury. There is significant tubular damage, characterized by tubular epithelial cell injury (cells appear flattened, swollen, or vacuolated, with potential loss of brush borders and focal necrosis/sloughing), tubular dilation, widening of the interstitial space (edema), increased infiltration of inflammatory cells within the interstitium, and possible presence of casts or debris within tubular lumens (Figure 6A).

Compared to the significant kidney damage observed in the CLP control group, both the treatment by R491 at both low and high doses shows significant improvement. R491 low-dose group shows a noticeable reduction in the severity of kidney damage compared to the CLP control group (Figure 6A). Specifically, there is less evidence of severe tubular epithelial injury (tubules appear more intact). Interstitial edema and inflammatory cell infiltration appear reduced, though still present compared to sham controls. The quantitative tubular damage score (Figure 6B) is significantly lower than the CLP control group, confirming partial protection. R491 high dose group exhibits even more pronounced protection against kidney injury compared to both the CLP control and the R491 lose dose groups (Figure 6A). The overall kidney architecture appears much better preserved, more closely resembling the Sham control. Tubular structures are largely intact with less evidence of cell damage, dilation, or casts. Interstitial edema and inflammation are further reduced compared to the low-dose group.

Both the histopathological images and quantitative analysis (Figure 6B) indicates that a CLP procedure induced substantial histopathological damage indicative of acute kidney injury and ALD-R491 administration dose-dependently attenuated the histopathological signs of acute kidney injury (tubular damage, interstitial edema, inflammation) induced by CLP sepsis.

In addition to the histopathological changes, renal functions were also measured by systemic levels of creatinine (CRE), a key biomarker of renal filtration function, and blood urea nitrogen (BUN), a key biomarker of renal function and protein metabolism. In sepsis, elevated CRE and BUN are hallmarks of acute kidney injury (AKI), and often signal hypoperfusion and metabolic imbalance. CLP-induced sepsis caused significant kidney dysfunction with elevated levels of both CRE and BUN. Treatment with ALD-R491 at both low and high doses significantly ameliorated this functional impairment (Figure 6C,D).

Consistent with the observed upregulation of inflammatory factors (*IL-1β*, *IL-6*, *TNF-α*) in the lungs and liver, the CLP group exhibited significantly elevated mRNA expression of these cytokines in the kidneys (Figure 6E–G). ALD-R491 demonstrated a dose-dependent inhibitory effect on the expression of these inflammatory genes (Figure 6E–G).

## 4. Discussion

Using cecal ligation and puncture (CLP) in mice, we demonstrated that targeting vimentin significantly reduced sepsis-related mortality. The orally administered vimentin modulator ALD-R491 dose-dependently reduced inflammation, protected multiple organs from injury, alleviated disease severity, and improved survival in CLP-induced sepsis. Whether administered prophylactically alone or therapeutically alongside broad-spectrum antibiotics, ALD-R491 significantly improved survival in a dose-dependent manner.

Sepsis-induced hyperinflammation is primarily driven by TNF-α, IL-6, and IL-1β, whose plasma levels negatively correlate with survival in patients [32]. TNF-α is one of the very first cytokines released by activated macrophages and monocytes, upon encountering microbial components, such as endotoxins. It triggers a cascade of events, including induction of IL-1 and IL-6, vascular alteration and organ damage. After endotoxin challenge, TNF-α appears first in circulation, peaking around 90 min [33], followed by IL-1, then IL-6 (peaking around 2–3 h), and subsequently acute-phase proteins like CRP [34]. Because systemic exposure of ALD-R491 peaked after 2 h following oral administration [23], the oral drug treatment at 2 h after the CLP procedure in this study represents a therapeutic intervention in the full-blown systemic inflammation.

Due to their central roles in driving harmful inflammation and downstream pathological cascades, TNF-α, IL-6, and IL-1 have been extensively investigated as therapeutic targets in sepsis and other cytokine storm-related conditions, such as severe COVID-19. Multiple agents targeting these pathways—including TNF-α or its receptor (e.g., infliximab, etanercept) [35], IL-6 or its receptor (e.g., sarilumab, tocilizumab) [36], and IL-1 (e.g., anakinra) [37]—have been evaluated in clinical trials. However, none have shown consistent survival benefit, and in some cases, potential harm was observed. In contrast, ALD-R491 demonstrated broad inhibition of all three key inflammatory cytokines in this study, potentially offering an advantage over therapies that target individual cytokines.

In sepsis, both metabolic disruption and cellular injury are key contributors to disease progression and organ failure, as reflected by elevated levels of lactate (Lac) and lactate dehydrogenase (LDH). Profound metabolic changes as the body shifts to meet the extreme energy demands of inflammation and infection. Lac is primarily produced through glycolysis, an anaerobic metabolic pathway. Although typically activated under low-oxygen conditions, sepsis triggers a profound stress response that accelerates glycolysis even in the presence of oxygen. In septic shock, tissue hypoperfusion further forces cells into anaerobic metabolism, leading to excessive lactate production. Additionally, mitochondrial dysfunction in sepsis impairs oxidative phosphorylation, diverting pyruvate toward lactate synthesis. Elevated lactate levels strongly correlate with disease severity and mortality risk. LDH, a ubiquitous intracellular enzyme that catalyzes the reversible conversion between pyruvate and lactate, is released into circulation upon cell damage. Increased serum LDH levels indicate widespread tissue injury and are associated with greater organ dysfunction and worse prognosis. In this study, ALD-R491 treatment dose-dependently reduced both Lac and LDH levels, suggesting mitigation of metabolic abnormalities and organ damage.

The lungs, liver, and kidneys are among the organs most commonly affected in sepsis [38]. ALD-R491 significantly attenuated acute injury in all three organs, as demonstrated by both biomarker analysis and histological evaluation. In treated mice, lung tissue showed reduced intra-alveolar exudate, interstitial edema, hemorrhage, and necrosis; liver sections exhibited decreased vascular congestion and inflammatory infiltration; and kidney tissues showed less tubular damage, interstitial edema, and inflammation. Systemic levels of ALT, AST, creatinine (CRE), and blood urea nitrogen (BUN)—biomarkers of hepatic and renal injury—were also significantly lowered by ALD-R491 treatment. These findings suggest that ALD-R491 exerts broad organ-protective effects, consistent with the wide-ranging activity of a vimentin modulator.

This study demonstrated that ALD-R491 effectively reduces hyperinflammation, organ injury, and mortality in septic mice; however, it was not designed to comprehensively evaluate the compound’s impact on other key pathogenic processes such as infection control, immunosuppression, and coagulopathy. Our previous studies, along with extensive research linking vimentin to these critical pathways, support the feasibility of vimentin modulation to simultaneously target multiple pathogenic components of sepsis.

Vimentin is known to facilitate microbial infections [11,12], and ALD-R491 has previously been shown to inhibit viral entry, trafficking, and egress, as well as to enhance bacterial clearance by macrophages [23]. In clinical practice, broad-spectrum antibiotics are administered to control infection immediately upon diagnosis. While bacteria are the primary cause, viruses, fungi, and parasites can also trigger sepsis. The condition progresses rapidly, often leaving insufficient time to identify the causative pathogen through blood cultures. Additionally, multidrug-resistant (MDR) bacterial infections are common in ICU patients, 30% of which are diagnosed with sepsis or develop it during their stay [39]. Although timely antibiotic administration is a cornerstone of sepsis management, the benefits and risks of broad-spectrum antibiotic use in all sepsis cases remain debated [40,41]. Modulating vimentin with ALD-R491 offers a host-directed antimicrobial strategy [23], which may be particularly useful for controlling infections caused by unknown or multidrug-resistant pathogens in sepsis.

As sepsis progresses, the immune response shifts from hyperinflammation to immunosuppression. During this phase, macrophages polarize toward the immunosuppressive M2 phenotype [42]; neutrophils become dysfunctional and lose their antimicrobial activity; effector T cells undergo exhaustion or functional impairment, while regulatory T cells (Tregs) become overproliferated and highly activated [43,44,45,46]. Vimentin expresses abundantly in both innate and adaptive immune cells and modulate their functions. Its involvement in inflammation is well documented in the literature, such as supporting NLRP3 inflammasome assembly and activation [14] and forming distal pole complex to restrain Treg functions. Vimentin also contributes to immune suppression. It is more highly expressed in M2 macrophages than in M1 macrophages and serves as a marker of the immunosuppressive phenotype [47]. In neutrophils, surface expression of vimentin filaments is a characteristic feature of apoptosis [48]. In lymphocytes, vimentin levels are elevated in patients with septic shock compared to non-shock cases and are associated with worse outcomes [20]. Collectively, these findings highlight vimentin’s involvement across key immune cell types and its potential role in driving the immunosuppressive state of late-stage sepsis. Modulating vimentin with ALD-R491 could not only dampen hyperinflammation, as demonstrated previously [28] and in this study, but also help prevent the onset of immunosuppression during disease progression.

The dual role of vimentin in both hyperinflammation and immunosuppression could present a unique opportunity for ALD-R491 to address the conflicting pathologies of sepsis, an area where other therapies have fallen short. Corticosteroids, although commonly used to suppress inflammation, remain controversial due to their immunosuppressive effects, which can exacerbate sepsis [49]. Furthermore, therapies that target single inflammatory mediators or isolated pathogenic components have proven ineffective against the rapidly evolving, multifactorial nature of sepsis. Our previous study showed that ALD-R491 can directly activate existing Treg cells by disassembling the distal pole complex [23] and completely block Treg cell proliferation in response to IL-2 and TGF-β (Aluda’s unpublished data). By modulating vimentin, ALD-R491 could offer the potential to inhibit both hyperinflammation and immunosuppression in sepsis.

Furthermore, vimentin contributes to sepsis-associated coagulopathy. By interacting directly with fibrinogen, it enhances fibrin clot formation [18]. Vimentin also facilitates platelet adhesion and aggregation through interactions involving both fibrinogen and von Willebrand factor (VWF) [50]. Additionally, septic endothelial cells promote a procoagulant state by upregulating surface adhesion molecules and releasing VWF, which interact with vimentin [17,50]. These mechanisms suggest that modulating vimentin could potentially counteract procoagulant activity during sepsis.

Although prior research has established roles for vimentin in regulating diverse cellular processes implicated in sepsis pathology, the present study was not designed to characterize these mechanisms in septic mice. The identification of vimentin as the specific target of ALD-R491 was reported previously and was not further explored here. The CLP-induced sepsis model remains valuable for testing novel therapeutics, yet its inherent variability limits precise control of disease severity across experiments, and no animal model can fully recapitulate the complexity of human sepsis. While ALD-R491’s mechanism of action addresses multiple, and sometimes opposing, pathophysiological processes of sepsis, and our studies were conducted under carefully controlled conditions to approximate clinical scenarios, translation to patients remains uncertain due to species differences, technical variability, limited biological heterogeneity, and the absence of standard supportive care.

In summary, vimentin is a unique target engaged in multiple aspects of sepsis pathogenesis. ALD-R491, the first oral small molecule specifically targeting vimentin, significantly reduced hyperinflammation, mitigated organ damage, and improved survival in the CLP-induced sepsis model. These therapeutic effects, together with previously characterized mechanisms of action, provide compelling preclinical proof-of-concept that vimentin targeting represents a promising strategy for developing effective sepsis therapeutics and warrants further clinical investigation.

## Data Availability

The datasets used and/or analyzed during the current study are available from the corresponding author on reasonable request.
